# PRF thermometry during MR-guided focused ultrasound ablation in a preclinical thiel model

**DOI:** 10.1186/2050-5736-3-S1-P49

**Published:** 2015-06-30

**Authors:** Ioannis Karakitsios, Martin Rube, Osnat Dogadkin, Senay Mihcin, Timur Saliev, Andreas Melzer

**Affiliations:** 1 University of Dundee, Dundee, United Kingdom

## Background/introduction

Proton Resonance Frequency (PRF) MR Thermometry is a useful method for treatment planning with MR-guided Focused Ultrasound (MRgFUS), as it provides accurate, real-time temperature maps. Thiel is an embalming medium that retains physical properties and life-like characteristics of human and animal tissue. The aim of the present study was to determine the accuracy of PRF Thermometry during MRgFUS and to estimate the value of PRF coefficient of pre-clinical Thiel embalmed human and animal tissue, and compare to fresh tissue and gel phantom.

## Methods

**Figure 1:**
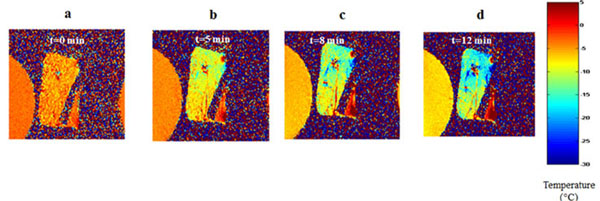
Reconstructed temperature maps of fresh ovine liver, heated in bulk, showing the tissue temperature during heating.

**Figure 2:**
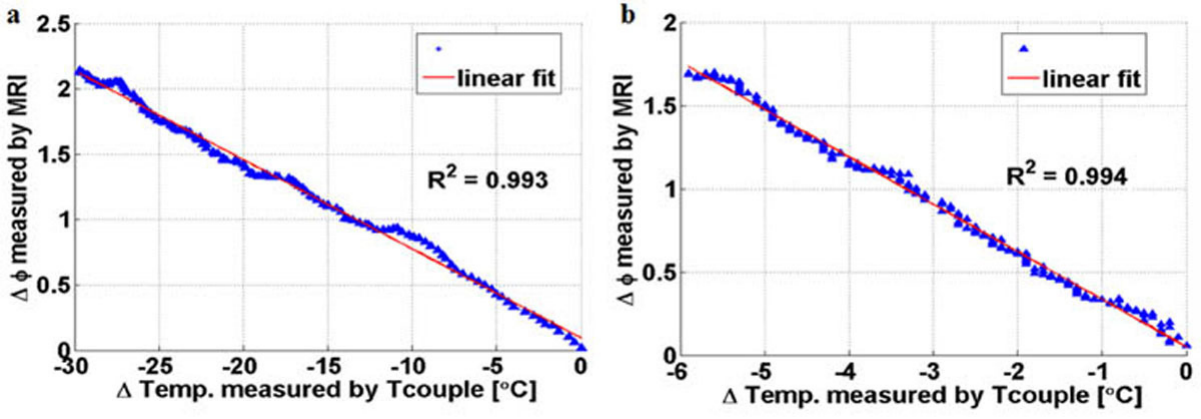
Example of graphs of the phase difference plotted as a function of temperature difference for: (a) bulk heating and (b) FUS-induced heating of Thiel embalmed human liver.

PRF Thermometry was conducted on Thiel embalmed human and animal liver during MRgFUS treatment on a FUS system (ExAblate 2000, InSightec, Tirat Carmel, Israel) embedded on a 1.5T scanner (Signa HDx, GE Medical Systems, Milwaukee, USA). The temperature rise based on PRF Thermometry during treatment was compared to the actual temperature increase measured by fibre optic thermocouples. To calculate the PRF shift coefficient, we applied phase-referenced PRF thermometry during cooling of the tissue, to obtain a series of phase difference, ΔΦ maps (Figure 1). The temperature difference, ΔΤ, was measured by thermocouples. The PRF shift coefficient was calculated from the measured ΔΦ,ΔΤ (Figure 2).

## Results and conclusions

**Figure 3:**
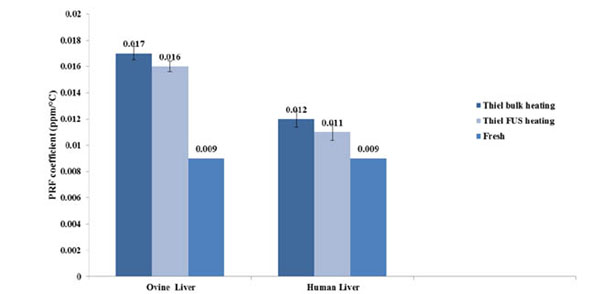
Graph of PRF shift coefficients of Thiel embalmed ovine and human liver heated with FUS or in bulk. The values of PRF coefficient in Thiel were plotted alongside with those for fresh tissue found in literature.

We found that the temperature differences and the PRF coefficient were higher for the Thiel organs than for fresh organs. This leads to the assumption that embalming a tissue with Thiel fluid can affect PRF Thermometry. The chemical composition of the Thiel fluid and the electrical conductivity might be some possible reasons for that. For the Thiel embalmed organs, we found temperature difference varying from 1.17C to 3.13C for ovine liver, and from 1.3C to 3.1C for human liver. For fresh tissue and phantom they were less than 0.4C. In the case of bulk heating, average values of PRF coefficient (±SD) were 0.016(4×10-4) ppm/C, 0.011(5×10-4) ppm/C for Thiel embalmed ovine liver and human liver, respectively (Figure 3).

